# Effects of Al-Impurity Type on Formation Energy, Crystal Structure, Electronic Structure, and Optical Properties of ZnO by Using Density Functional Theory and the Hubbard-U Method

**DOI:** 10.3390/ma9080647

**Published:** 2016-08-01

**Authors:** Hsuan-Chung Wu, Hsing-Hao Chen, Yu-Ren Zhu

**Affiliations:** Department of Materials Engineering, Ming Chi University of Technology, New Taipei 24301, Taiwan; m02188017@mail2.mcut.edu.tw (H.-H.C.); ken_11357@yahoo.com.tw (Y.-R.Z.)

**Keywords:** density functional theory, first-principles calculations, electronic structure, optical property, Al-impurity, ZnO

## Abstract

We systematically investigated the effects of Al-impurity type on the formation energy, crystal structure, charge density, electronic structure, and optical properties of ZnO by using density functional theory and the Hubbard-U method. Al-related defects, such as those caused by the substitution of Zn and O atoms by Al atoms (Al_s(Zn)_ and Al_s(O)_, respectively) and the presence of an interstitial Al atom at the center of a tetrahedron (Al_i(tet)_) or an octahedron (Al_i(oct)_), and various Al concentrations were evaluated. The calculated formation energy follows the order E_f_(Al_s(Zn)_) < E_f_(Al_i(tet)_) < E_f_(Al_i(oct)_) < E_f_(Al_s(O)_). Electronic structure analysis showed that the Al_s(Zn)_, Al_s(O)_, Al_i(tet)_, and Al_i(oct)_ models follow *n*-type conduction, and the optical band gaps are higher than that of pure ZnO. The calculated carrier concentrations of the Al_s(O)_ and Al_i(tet)_/Al_i(oct)_ models are higher than that of the Al_s(Zn)_ model. However, according to the curvature of the band structure, the occurrence of interstitial Al atoms or the substitution of O atoms by Al atoms results in a high effective mass, possibly reducing the carrier mobility. The average transmittance levels in the visible light and ultraviolet (UV) regions of the Al_s(Zn)_ model are higher than those of pure ZnO. However, the presence of an interstitial Al atom within the ZnO crystal reduces transmittance in the visible light region; Al_s(O)_ substantially reduces the transmittance in the visible light and UV regions. In addition, the properties of ZnO doped with various Al_s(Zn)_ concentrations were analyzed.

## 1. Introduction

Transparent conductive oxides (TCOs) are crucial in the photoelectric industry. They can be used in photoelectric applications, such as tablet PCs [1], light-emitting diodes [2], and solar cells [3]. High transmittance (>80%) and low resistivity (<10^−3^ Ω·cm) are necessary conditions for TCOs to operate efficiently [4]. ZnO with a wide band gap exhibits high transmittance in the visible light region and is a potential alternative material for indium tin oxide [5]. Although intrinsic defects, such as oxygen vacancies and interstitial Zn atoms, cause ZnO to exhibit *n*-type conduction, they are unstable or cannot supply conductive electrons at room temperature. Doping ZnO thin film with impurities is an effective method for improving the electric characteristics of ZnO [6,7,8].

Among various types of doped ZnO, Al-doped ZnO (AZO) is inexpensive, possesses excellent electric and optical properties, and has been extensively researched. In addition, the synthesis of AZO by low-cost, low-temperature techniques such as chemical bath deposition [9,10] has been achieved recently [9,10]. Agura et al. [11] fabricated a series of AZO thin films by using pulsed laser deposition and achieved a resistivity of 8.54 × 10^−5^ Ω·cm and an average transmittance higher than 88% in the visible light region. Fragala et al. [12] indicated that the carrier concentration and optical band gap of AZO increase with an increase in the Al concentration. Maeng et al. [13] fabricated AZO by using atomic layer deposition and various Al doping concentrations from 1 to 12 at %, showing that the transmittance increases with an increase in the Al concentration. Blagoev et al. [14] also prepared AZO films with different Al concentrations by atomic layer deposition. They found that the resistivity of the films decreased with an increase in the Al concentration, reaching a minimum of 3.3 × 10^−3^ Ω·cm at about 1.1% Al_2_O_3_ and then increased slowly. Singh et al. [15] found the existence of shallow donor-level defects in AZO films directly contributes to the carrier concentrations, whereas deep donor-level defects were not found to contribute to the carrier concentrations. In addition, AZO thin films were prepared using sol-gel spin coating and various Al concentrations (1–5 wt %) [16]. The results showed that the unit cell volume of AZO decreases with an increase in the Al concentration, which may be attributed to the substitution of Al^3+^ atoms (small ionic radius) with Zn^2+^ (large ionic radius). In addition, the *c*-axis lattice parameter decreases from 1 to 4 wt %. Furthermore, Periasamy et al. [17] observed that the c-axis lattice constant increases from 0.52052 to 0.52422 nm with an increase in the Al concentration from 0% to 6%, which is attributed to the incorporation of Al^3+^ ions in the interstitial positions. Regarding theoretical calculations of AZO properties, Qu et al. [18] used the CASTEP software to analyze the thermoelectric properties and electronic structure of AZO. Palacios et al. [19] used the density functional theory and Hubbard-U method (DFT+U method) to correct band-gap underestimation. They used the optimal effective Hubbard-U (U_eff_) of 8.5 eV and calculated the ZnO band gap (2.13 eV), which was lower than the experimental value of 3.4 eV. Gabás et al. [20] indicated the decrease in the AZO film resistivity is due to the filling of the Al impurity band state by using the DFT+U method. According to the calculated results regarding the formation energy, Li et al. [21] indicated that a single doping of Al forms easily, particularly at the extreme O-rich limit. In addition, calculations of the effective mass showed that a single doping of Al has a low effective mass.

Although theoretical calculations of AZO structures were performed, most conventional DFT calculations highly underestimate the ZnO band gap. In our previous study [22], we used the DFT+U method to avoid underestimating the ZnO band gap. In this study, we extended the DFT+U method to systematically analyze the formation energy, crystal structure, electronic structure, and optical properties of AZO structures. The theoretical calculation results are expected to facilitate future material design.

## 2. Calculation Methods

**Figure 1:**
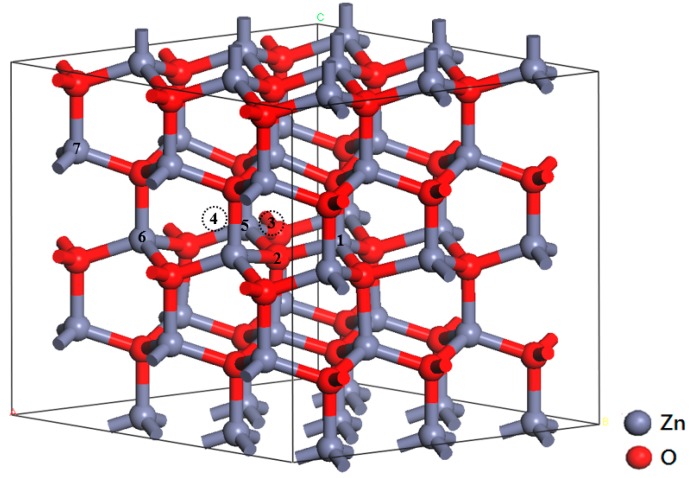
A 3 × 3 × 2 supercell containing substitutional and interstitial Al atoms. Gray and red spheres represent Zn and O atoms, respectively; 1–7 represent the locations of substituted and interstitial (dotted line circle) Al atoms.

To systematically analyze the properties of various AZO structures, a 3 × 3 × 2 supercell containing 36 Zn atoms and 36 O atoms was used (Figure 1). Four structures, one in which Zn atoms are substituted by Al atoms (Al_s(Zn)_), one in which O atoms are substituted by Al atoms (Al_s(O)_), one in which an interstitial Al atom is present in a tetrahedron (Al_i(tet)_), and one in which an interstitial Al atom is present in an octahedron (Al_i(oct)_), were examined and labeled 1, 2, 3, and 4, respectively. For calculating the Al concentration, models with one (Al_s(Zn)_), two (2Al_s(Zn)_), and three (3Al_s(Zn)_) Al atoms at the substitutional Zn sites, corresponding to Al concentrations of 2.78, 5.56, and 8.33 at %, respectively, were analyzed.

All calculations were performed using the CASTEP code [23] based on DFT. Ion cores were modeled using ultrasoft pseudopotentials [24]. The valence configurations of the Zn, O, and Al atoms were 4s^2^3d^10^, 2s^2^2p^4^, and 3s^2^3p^1^, respectively. The cutoff energy of the plane wave was 380 eV. The Monkhorst-Pack k-point was 4 × 4 × 2 [25]. Structure optimization was conducted before property calculation. For structure optimization, the exchange and correlation interactions were calculated using the generalized gradient approximation function. In structure optimization calculations, the energy change, maximum force, maximum stress, and maximum displacement were fixed at 10^−5^ eV/atom, 0.03 eV/Å, 0.05 GPa, and 0.001 Å, respectively. For calculating the properties, we used the DFT+U_d_+U_p_ method, in which the *U_d_* value for Zn-3d and the *U_p_* value for O-2p orbitals were set at 10 and 7 eV, respectively [26]. Differences in the band structures, band gaps, and Zn-3d orbital locations of pure ZnO for various *U_d_* and *U_p_* values can be referred to in our previous study [27].

## 3. Results and Discussion

### 3.1. Formation Energy

To determine the relative stability for various AZO models, the formation energy was calculated as follows.

(1) $$E_{f}({\mathrm{Al}}_{s,i})=E_{\mathrm{defect}}({\mathrm{Al}}_{s,i})-[E_{\mathrm{perfect}}(\mathrm{ZnO})-{L\mu }_{\mathrm{Zn}}-{M\mu }_{o}+{N\mu }_{\mathrm{Al}}]$$

where E_f_(Al_s,i_) and E_defect_(Al_s,i_) represent the formation energy and total energy for substitutional and interstitial Al defects, respectively; E_perfect_(ZnO) is the total energy of a perfect ZnO supercell; L, M, and N are the numbers of substitutional or interstitial Al atoms; and μ is the chemical potential of various atoms. The formation energy is related to the growth atmosphere, which can be divided into O-rich and Zn-rich conditions. For ZnO, μ_Zn_ and μ_O_ satisfy the relation μ_Zn_ + μ_O_ = μ_ZnO_. Under O-rich conditions, μ_O_ is half of the total energy of an O_2_ molecule. Under Zn-rich conditions, μ_Zn_ and μ_Al_ are the energies of one Zn atom in bulk Zn and one Al atom in bulk Al, respectively.

Table 1 summarizes the calculated formation energy of various types of AZO. The results show that regardless of whether conditions are O-rich or Zn-rich, the formation energy follows the order E_f_(Al_s(Zn)_) < E_f_(Al_i(tet)_) < E_f_(Al_i(oct)_) < E_f_(Al_s(O)_). This means that Al atoms most likely replace Zn atoms, followed by interstitial sites, and they least likely replace O atoms. The occupancy of interstitial sites by Al atoms was observed in a previous study [17]. In addition, E_f_(Al_s(Zn)_) is lower under O-rich conditions than under Zn-rich conditions, showing that an O-rich atmosphere is easier to form in the Al_s(Zn)_ structure. The calculated formation energy reported by Li et al. [21] follows the same trend.

To evaluate the effect of the Al concentration, we performed structure optimization by varying the distance between two Al dopant atoms. We fixed one Al atom on the number 1 site and another Al atom on the number 5 (near), 6 (medium), and 7 (far) sites (Figure 1). The results show that the total energy of the structure with the short distance is the highest. The energies of supercells obtained using the medium and long distances are lower (0.25 and 0.3 eV, respectively) than that obtained using the short distance, suggesting that Al atoms tend to disperse in ZnO. Therefore, we retained the long distance between the two impure Al atoms.

### 3.2. Crystal Structure

Table 2 summarizes the lattice parameters, unit cell volume, and average bond lengths after structure optimization. For the Al_s(Zn)_ structure, the length of the Al–O bond (1.797 Å) is shorter than that of the Zn–O bond (1.997 Å) in pure ZnO, resulting in shrinkage in cell volume. The shrinkage of the Al–O bond may be caused by the difference in the radii of the ions (0.51 Å for Al^3+^ and 0.74 Å for Zn^2+^). In addition, comparing the pure ZnO, the Al_s(Zn)_, 2Al_s(Zn)_, and 3Al_s(Zn)_ models revealed that the cell volume decreases with an increase in the Al_s(Zn)_ concentration, which is consistent with previously reported results [16].

**Figure 2:**
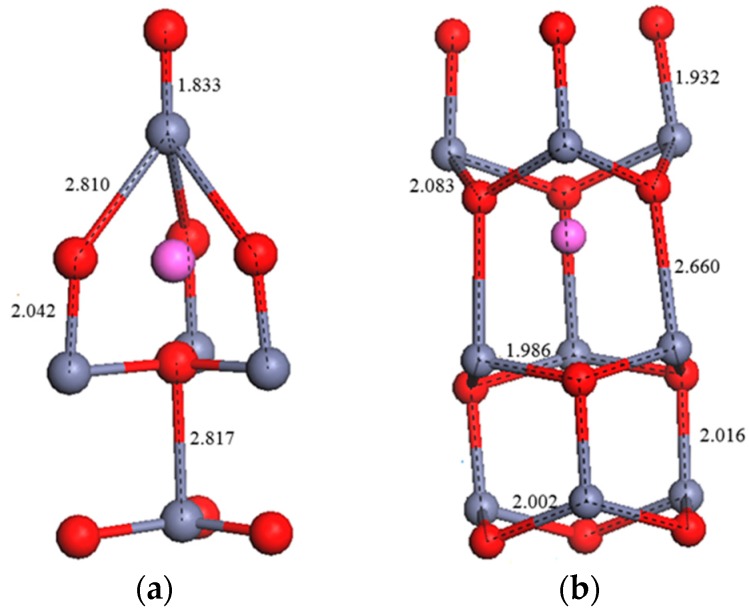
Optimized structures of interstitial Al atoms in (**a**) tetrahedron and (**b**) octahedron locations after geometry optimization.

Conversely, when one O atom is substituted with one Al atom (Al_s(O)_ structure), the repulsive force between the Al and Zn ions leads to an increase in the lengths of the Zn–O and Al–Zn bonds and an expansion in volume. The presence of interstitial Al atoms leads to an increase in the *c*-axis lattice constant and the expansion of the cell volume. The Zn–O bonds around the interstitial Al atoms in the tetrahedron and octahedron locations after geometry optimization are shown in Figure 2. Periasamy et al. [17] indicated that the *c*-axis lattice constant increases from 5.205 to 5.242 Å with an increase in the Al concentration from 0% to 6%. This was attributed to the incorporation of Al^3+^ ions in interstitial positions.

### 3.3. Charge Density

**Figure 3:**
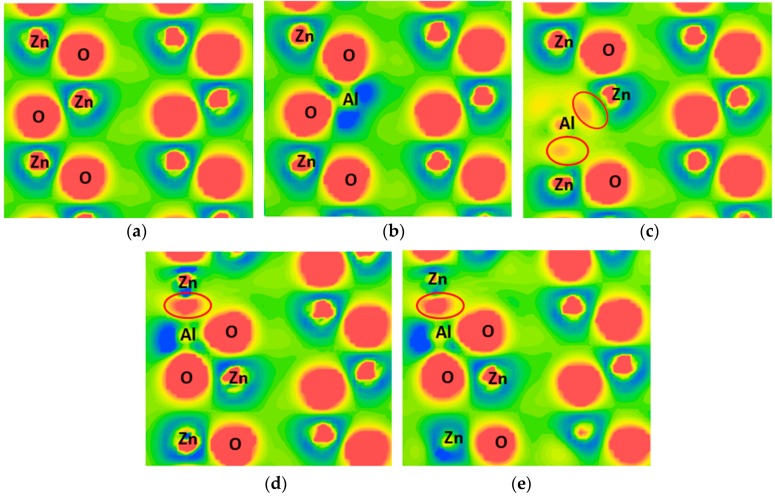
Distribution of charge density difference for (**a**) pure ZnO; (**b**) Al_s(Zn)_; (**c**) Al_s(O)_; (**d**) Al_i(tet)_; and (**e**) Al_i(oct)_.

Mulliken atomic population and bond population analysis was used to describe the charge transfer and the bond type after bonding, respectively. Table 3 summarizes the Mulliken population of each structure. Positive and negative values of the atomic population represent the atom losing and gaining electrons, respectively. A high bond population is characteristic of a covalent bond; conversely, a low bond population is characteristic of an ionic bond. In addition, contour plots of the difference in the charge density associated with AZO structures are shown in Figure 3, in which a higher value (red) represents gaining electrons.

For the Al_s(Zn)_ structure, Table 3 shows that the atomic population of Al (1.62) is higher than that of Zn (0.94) because of differences in valence electrons between Al and Zn atoms. The populations of O atoms in the Al_s(Zn)_ and pure ZnO structures are −1.02 and −0.94, respectively, indicating that numerous electrons are transferred from Al atoms to O atoms. In addition, the population of the Al–O bond (0.5) is higher than that of the Zn–O bond (0.39). This implies that the covalent characteristic of the Al–O bond is high, which is consistent with the qualitative analysis results (Figure 3b). The high covalent characteristic of Al–O is stable, which may be the reason that the formation energy of the Al_s(Zn)_ model is negative. The populations of O and Al atoms do not change with an increase in the Al concentration (Al_s(Zn)_, 2Al_s(Zn)_, and 3Al_s(Zn)_), except for a slight reduction in the population of Zn atoms.

In the Al_s(O)_ structure, Al atoms gain an electron from the adjacent Zn atoms (Al atomic population is negative). In addition, Al and Zn form a covalent bond (0.94). The share charge is shown in Figure 3c. For the Al_i(tet)_ and Al_i(oct)_ models, the atomic population of Al and the bond population of the Al–O bond are lower than those of the Al_s(Zn)_ model. The share charge is also observed in Figure 3d,e.

### 3.4. Electric Properties

**Figure 4:**
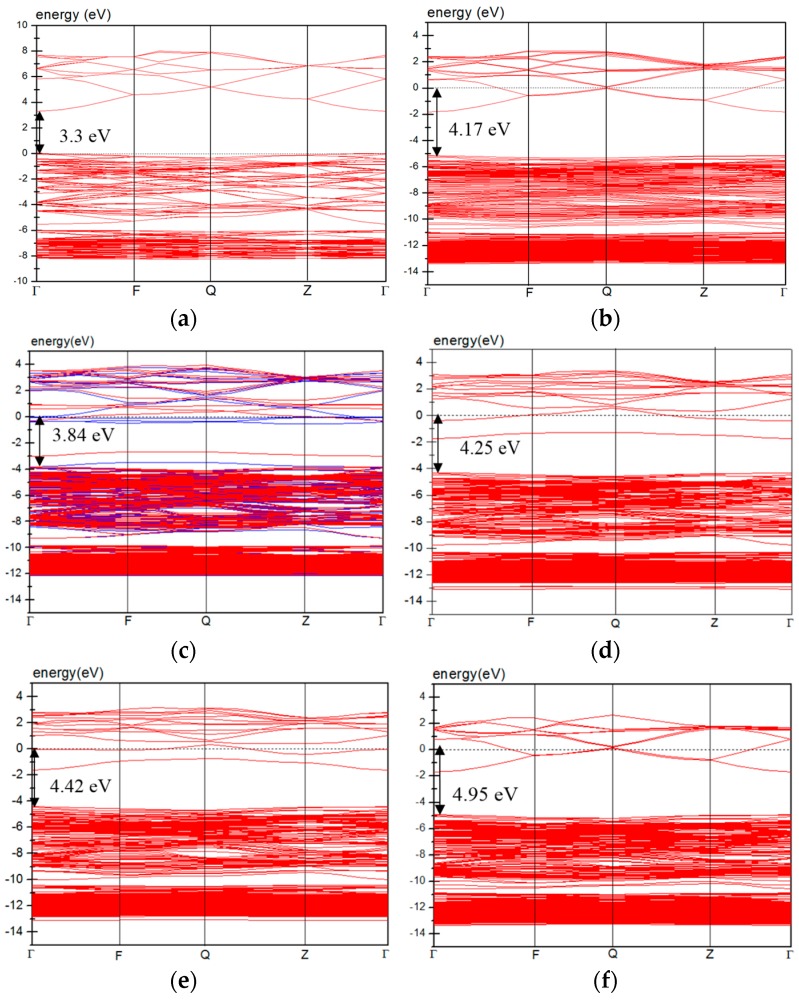

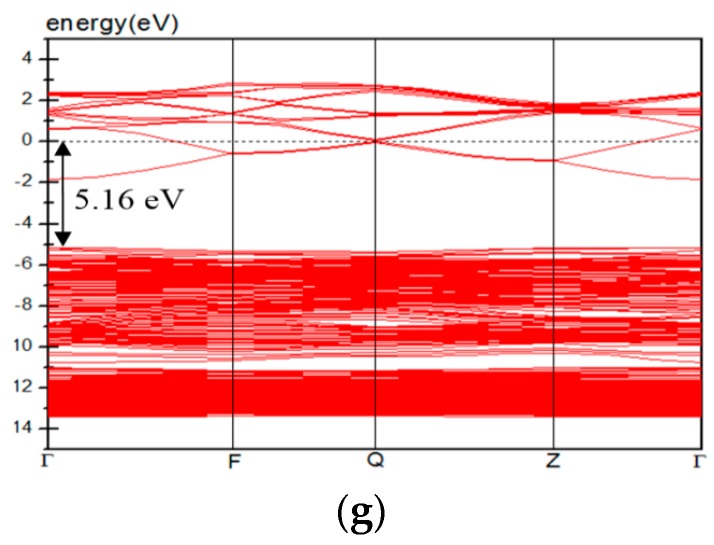
Band structures of (**a**) pure ZnO; (**b**) Al_s(Zn)_; (**c**) Al_s(O)_; (**d**) Al_i(tet)_; (**e**) Al_i(oct)_; (**f**) 2Al_s(Zn)_; and (**g**) 3Al_s(Zn)_.

Figure 4 shows the band structures of pure ZnO and AZO. The energy zero (eV) indicated by a dotted line is the Fermi level. Figure 4a indicates that pure ZnO is a direct-gap semiconductor material with a band gap of 3.3 eV, which is consistent with the experimental value.

After the substitution of a Zn atom by an Al atom, shallow donor states form at the bottom of the conduction band because the valence of Al exceeds that of Zn. Therefore, the band structure of the Al_s(Zn)_ model exhibits an *n*-type characteristic with an optical band gap of 4.17 eV (Figure 4b). In addition, the optical band gap increases with an increase in the Al_s(Zn)_ concentration (Figure 4f,g). The same trend was observed in a previous experimental study [12] and hybrid functional calculations [28]. 

The Al_s(O)_, Al_i(tet)_, and Al_i(oct)_ models (Figure 4c,d) show *n*-type conductive characteristics, and the optical band gaps of all of these models are higher than that of pure ZnO. Except for the donor states, deep donor states form in the band gap in the three models. 

In the band structure, the curvature closer to the bottom of the conduction band affects the size of the electron effective mass. A flatter band with a smaller curvature results in a higher effective mass of electrons in the conduction band. A high effective mass of electrons is related to low carrier mobility and reduced electrical conductivity. Comparing the occupied energy level at the Γ point near the Fermi energy revealed that the energy level curvatures of the pure ZnO and Al_s(Zn)_ models (including 2Al_s(Zn)_ and 3Al_s(Zn)_ models) are higher than those of the Al_s(O)_, Al_i(tet)_, and Al_i(oct)_ models. This implies that the occurrence of interstitial Al atoms or the substitution of O atoms by Al atoms results in a high effective mass, and this decreases the carrier mobility and electric conductivity.

**Figure 5:**
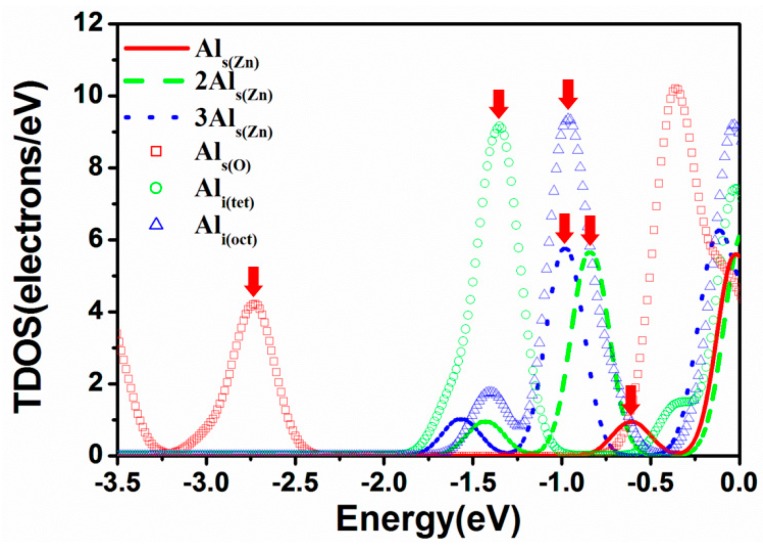
TDOS near Fermi energy for AZO models.

Figure 5 shows the portion of total density of states (TDOS) below the Fermi energy (E_F_, 0 eV) for various AZO models. In general, the occupied states close to the Fermi energy contribute numerous free electrons to the conduction band, and the occupied states far from the Fermi energy do not easily supply free electrons to the conduction band. We divided the TDOS of each model into two parts, close to E_F_ and far from E_F_ (indicated by a red arrow shown in Figure 5), and integrated each part to evaluate the carrier concentration. Table 4 shows the calculated carrier concentration. The total carrier concentration for the Al_s(Zn)_ model is 12.5 × 10^20^ #/cm^3^, and the carrier concentrations close to E_F_ and far from E_F_ are 9.7 × 10^20^ and 2.8 × 10^20^ #/cm^3^, respectively. As expected, the total carrier concentration increases from 12.5 × 10^20^ to 33.5 × 10^20^ of 3Al_s(Zn)_ #/cm^3^ with an increase in the Al_s(Zn)_ concentration. However, the concentration close to E_F_ decreases from Al_s(Zn)_ (9.7 × 10^20^ #/cm^3^) to 2Al_s(Zn)_ (7.9 × 10^20^ #/cm^3^) and increases from 2Al_s(Zn)_ to 3Al_s(Zn)_ (15.9 × 10^20^ #/cm^3^). This is because more occupied states contribute to the carrier concentration far from E_F_. Therefore, we suggest that the concentration of free electrons in experimental measurements does not absolutely increase with an increase in the Al concentration. In addition, the order of the calculated carrier concentrations is consistent with that of previously reported experimental results [11,12].

Both carrier concentrations close to E_F_ and far from E_F_ for the Al_s(O)_, Al_i(tet)_, and Al_i(oct)_ models are higher than those for the Al_s(Zn)_ model. This implies that substitution of an O atom by an Al atom or the presence of an interstitial Al atom increases the concentration of free electrons.

### 3.5. Optical Properties

To investigate the photoabsorption properties of the AZO system, calculating the imaginary part of the dielectric function ε_2_ (ω) is essential [29]:

(2) $$\varepsilon_{2}=\frac{2e^{2}\pi }{{\Omega \varepsilon }_{0}}\underset{k,v,c}{\sum }│〈\varphi_{k}^{c}│u\cdot r│\varphi_{k}^{v}〉{│}^{2}\delta (E_{k}^{c}-E_{k}^{v}-\omega )$$

where e is the electric charge, Ω is the unit cell volume, u is the polarization vector of the incident electric field, ω is the frequency of light, and $\varphi_{k}^{v}\mathrm{and}\varphi_{k}^{c}$ are the wave functions of the conduction and valence bands, respectively. Absorption and reflection coefficients can be obtained using the real and imaginary parts of the dielectric function. The relationship between thin-film thickness and transmittance can be written as follows:

(3) $$T={\left(1-R\right)}^{2}e^{-\alpha d}$$

where T is the transmittance; R and α are the reflection and absorption coefficients, respectively; and d is the thin-film thickness, which is assumed to be 250 nm.

**Figure 6:**
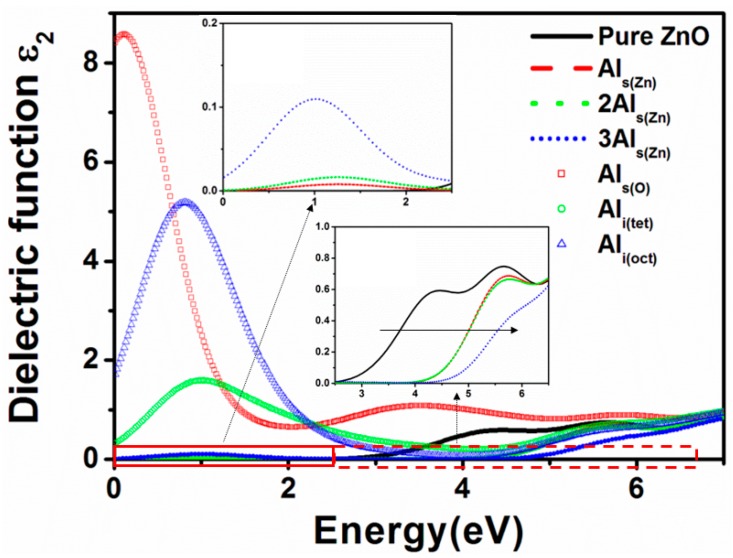
Imaginary part of the dielectric function for AZO models.

Figure 6 shows the calculated imaginary part ε_2_ (ω) of the dielectric function, and Table 5 lists the calculated average transmittance in the visible light region (400–800 nm) and ultraviolet (UV) region (200–400 nm). For the Al_s(Zn)_ model, the peak at 1.3 eV (left inset in Figure 6) is due to the shallow donor state, resulting in slight absorption in the long wavelength region (infrared and visible light). With an increase in the Al_s(Zn)_ concentration, the peak slightly enlarges. As mentioned in Section 3.4, the optical band gap increases with an increase in the Al_s(Zn)_ concentration. The increased optical band gap of the Al_s(Zn)_ model shifts the intrinsic absorption edge (blue shift; right inset in Figure 6) and significantly increases the transmittance (Table 5). According to the experimental results of Maeng et al. [13], the transmittance of AZO increases with an increase in the Al concentration. In addition, Fan et al. [28] also showed that the absorption decreases in the UV region and increases in the near-IR region with an increase in the Al doping concentration, which is similar to the trend in this study.

The peaks near 1 eV for the Al_i(tet)_ and Al_i(oct)_ models are stronger and broader than that of the Al_s(Zn)_ model and are mainly contributed by the shallow and deep donor states described in Section 3.4. These two peaks of the Al_i(tet)_ and Al_i(oct)_ models result in a significant reduction in the transmittance in the visible light region to 73.5% and 68.5%, respectively. However, the increased optical band gaps of the Al_i(tet)_ and Al_i(oct)_ models result in a blue shift of the intrinsic absorption edge and an increase in the transmittance in the UV region. Among all models, the red peak for the Al_s(O)_ model (Figure 6) is the strongest and widest because more impurity states (shallow and deep donor states) are distributed in the band gap. Therefore, regardless of the visible light or UV light region, the Al_s(O)_ model exhibits low transmittance (Table 5). The presence of an interstitial Al atom or the substitution of an O atom by an Al atom is expected to significantly reduce the transmittance.

## 4. Conclusions

This study systematically investigated the formation energy, crystal structure, charge density, electronic structure, and optical properties of ZnO with various types of Al-related defects by using the DFT+U method. The calculated formation energy indicated that in AZO preparations, the most probable structures are those in which Zn atoms are substituted by Al atoms and those involving interstitial Al atoms. For the Al_s(Zn)_ model, the covalence of the Al–O bond is greater than that of the Zn–O bond. The cell volume slightly decreases and the optical band gaps increase with an increase in the Al_s(Zn)_ concentration, resulting in high transmittance in the UV region. For evaluating the carrier concentration, the DOS area was integrated, and the Al_s(O)_, Al_i(tet)_, and Al_i(oct)_ models exhibit a higher carrier concentration than that of the Al_s(Zn)_ model. However, the Al_s(O)_, Al_i(tet)_, and Al_i(oct)_ models have a high effective mass compared with the Al_s(Zn)_ model. In addition, the presence of the Al_i(tet)_ model, the Al_i(oct)_ model, and particularly the Al_s(O)_ model within a ZnO crystal structure significantly reduces the transmittance in the visible light region. Therefore, the relation between structure and property for AZO can be a reference for adjusting the process parameters to fabricate TCO films.

## Figures and Tables

**Table 1:** Formation energy of AZO.

Models	Formation Energy (eV)	
	O-Rich	Zn-Rich
Al_s(Zn)_	−6.69	−3.45
Al_s(O)_	9.19	5.95
Al_i(tet)_	1.81	1.81
Al_i(oct)_	2.97	2.97

**Table 2:** Optimized lattice constants, bond length, and cell volume of AZO.

Models	Lattice Constants			Volume	Bond Length (Å)		
	a (Å)	c (Å)	c/a	unit cell (Å)^3^	Zn–O	Al–O	Al–Zn
Pure ZnO	3.282	5.265	1.60	49.01	1.997	---	---
Al_s(Zn)_	3.280	5.269	1.61	48.98	2.002	1.797	---
Al_s(O)_	3.304	5.329	1.61	50.21	2.009	---	2.451
Al_i(tet)_	3.283	5.392	1.64	50.56	2.015	1.855	2.286
Al_i(oct)_	3.286	5.418	1.65	50.27	2.023	1.803	2.695
2Al_s(Zn)_	3.278	5.271	1.61	48.92	2.009	1.797	---
3Al_s(Zn)_	3.274	5.275	1.61	48.87	2.015	1.796	---

**Table 3:** Atomic and bond population of AZO.

Models	Atomic Population (|e|)			Bond Population (|e|)		
	Zn	O	Al	Zn–O	Al–O	Al–Zn
Pure ZnO	0.94	−0.94	---	0.4	---	---
Al_s(Zn)_	0.94	−1.02	1.62	0.39	0.5	---
Al_s(O)_	0.92	−0.93	−0.44	0.39	---	0.94
Al_i(tet)_	0.90	−0.94	1.41	0.38	0.44	0.46
Al_i(oct)_	0.91	−0.93	0.90	0.38	0.39	0.42
2Al_s(Zn)_	0.93	−1.02	1.62	0.38	0.49	---
3Al_s(Zn)_	0.91	−1.02	1.63	0.37	0.50	---

**Table 4:** Carrier concentration of AZO calculated using DOS.

Models	Carrier Concentration (10^20^/cm^3^)		
	Total	Close to E_F_	Far from E_F_
Al_s(Zn)_	12.5	9.7	2.8
Al_s(O)_	46.9	32.8	14.1
Al_i(tet)_	32.7	10.8	21.9
Al_i(oct)_	32.8	10.9	21.9
2Al_s(Zn)_	25.1	7.9	17.2
3Al_s(Zn)_	33.5	15.9	17.6

**Table 5:** Average transmittance of AZO in UV and visible light regions.

Models	Transmittance (%)	
	UV	Visible Light
Pure ZnO	64.4	88.8
Al_s(Zn)_	75.2	91.1
Al_s(O)_	50.9	70.4
Al_i(tet)_	69.7	73.5
Al_i(oct)_	75.3	68.5
2Al_s(Zn)_	75.5	91.1
3Al_s(Zn)_	81.1	91.2
